# 

**DOI:** 10.1055/s-0035-1570111

**Published:** 2016-01

**Authors:** Caroline San Severino Teixeira, Antônio Carlos Vieira Cabral

**Affiliations:** 1 Programa de Pós-Graduação em Saúde da Mulher (Mestrado) da Universidade Federal de Minas Gerais, Belo Horizonte, MG, Brasil; 2 Faculdade de Medicina da Universidade Federal de Minas Gerais, Belo Horizonte, MG, Brasil

**Keywords:** gestação, hábitos alimentares, nutrição pré-natal, estado nutricional, ganho de peso, pregnancy, eating habits, prenatal nutrition, nutritional status, weight gain

## Abstract

**Objetivo** Verificar diferenças em alguns aspectos nutricionais de gestantes acompanhadas em serviço de atenção pré-natal em uma cidade do interior e na região metropolitana.

**Métodos** Foram avaliadas gestantes em atendimento pré-natal na cidade de Belo Horizonte (BH), região metropolitana, e Paula Cândido (PC), interior de MG. Aplicou-se um Questionário de Frequência Alimentar (QFA) contendo informações socioeconômicas e sobre o hábito alimentar, além disso, foram aferidos peso e altura no momento do atendimento e questionado o peso pré-gestacional, para posterior cálculo do IMC (índice de massa corpórea). A análise dos dados foi dividida por região e trimestre gestacional, utilizando o software SPSS versão 15.0, teste t para comparação de médias e qui-quadrado de independência, com 5% de significância.

**Resultados** Foram incluídas 240 gestantes, sendo 90 do interior e 150 da região metropolitana. Destas, a maioria são casadas (BH = 56,6%; PC = 46,6%), não trabalham fora de casa (BH = 54,6%; PC = 84,4%), predominantemente se alimentam 3 a 4 vezes ao dia no 1° e 2° trimestre (BH = 54,0 e 46,0%; PC = 66,7 e 63,3%, respectivamente) e fazem 5 a 6 refeições ao dia no 3° trimestre em BH (44%). Houve ganho de peso significativo somente no 1° trimestre (BH: 58,0%; PC: 53,33%). Ganho de peso versus hábito alimentar foi significativo para as variáveis “almoça ou janta fora de casa,” no 1° trimestre BH ( *p* = 0,006); “quantas vezes consome leite,” no 1° trimestre PC( *p* = 0,03); “quantas vezes consome *fastfood* ,” no 3° trimestre BH ( *p* = 0,009).

**Conclusões** As gestantes em ambas regiões se alimentam de forma adequada, apesar da prevalência de sobrepeso pré-gestacional em BH e baixo nível de escolaridade e renda, principalmente no interior, indicador que pode ser pouco favorável à nutrição das gestantes neste período. Estudos de associação entre hábito alimentar e saúde do recém-nascido irão contribuir para maiores informações sobre a nutrição no período gestacional.

## Abstract (en)

**Objective** To determine differences in some nutritional aspects of pregnant women assisted at prenatal care services in a country town and in a metropolitan area.

**Methods** Pregnant women received prenatal care in the city of Belo Horizonte (BH), metropolitan area, and Paula Cândido (PC), a country town. A Food Frequency Questionnaire (FFQ) containing socioeconomic information and information about eating habits was applied. In addition,weight and height were measured on the occasion of the visits and the women were ask to give their prepregnancy weight for subsequent BMI calculation. Data were analyzed according to region and trimester of pregnancy using the SPSS software version 15.0, the *t* -test to compare averages and the chi-square test of independence, with the level of significance set at 5%.

**Results** 240 pregnant women were included, 90 from the country town and 150 from the metropolitan area. Of these, most were married (BH = 56.6%; PC = 46.6%) and did not work outside the home (BH = 54.0%; PC = 84.4%). They predominantly had 3–4 meals/ day during the 1 ^st^ and 2 ^nd^ quarters (BH = 54.0 and 46%; PC = 66.7 and 63.3%, respectively) and had 5–6 meals/day during Q3 in BH (44%). There was significant weight gain only in the 1st quarter (BH: 58,0%; PC: 53.3%). Weight gain versus eating habits was significant for the variables “lunch or dinner away from home” for the 1 ^st^ quarter in BH ( *p* = 0.006), “How many times they consume milk” in the 1 ^st^ quarter in PC ( *p* = 0.03), and “How many times they consume junk food” in the 3 ^rd^ quarter in BH ( *p* = 0.009).

**Conclusions** Pregnant woman showed proper eating habits in both regions despite the prevalence of pregestational overweight in BH and a low level of education and income, especially in the country town, an indicator that may be unfavorable for the nutrition of pregnant women during this period. Studies of association between eating habits and newborn health will provide more information about nutrition during pregnancy.

## Introdução

A gestação é um período que impõe necessidades nutricionais aumentadas, e a adequada nutrição é primordial para a saúde da mãe e do feto. Gestantes devem consumir alimentos em variedade e quantidade específicas, considerando as recomendações dos guias alimentares e as práticas alimentares culturais, para atingir as necessidades energéticas e nutricionais, e as recomendações de ganho de peso. 1

As gestantes são suscetíveis à inadequação nutricional, pelo aumento da demanda de energia, macro e micronutrientes, que ocorrem durante a gravidez. A qualidade da alimentação e o estado nutricional da mulher, antes e durante a gravidez, afetam o crescimento e o desenvolvimento fetal, bem como a evolução da gestação. 2

A inadequação do ganho de peso durante a gestação tem sido apontada como fator de risco tanto para a mãe quanto para o concepto. 3 O ganho de peso aquém do recomendado pode acarretar restrição de crescimento intrauterino, parto prematuro, baixo peso ao nascer e aumento das taxas de morbimortalidade perinatal. 4 5 O ganho excessivo está associado, no feto, a, macrossomia, desproporção céfalo-pélvica, asfixia e na mãe, diabetes mellitus gestacional, hipertensão arterial, pré-eclâmpsia, eclampsia, maior retenção de peso pós-parto e aumento do risco de obesidade futura. 6 7

Neste sentido, é fundamental dispor de instrumentos capazes de avaliar a ingestão alimentar materna de forma a identificar, com eficácia e precisão, associações diretas entre a alimentação e a saúde da mãe e do feto. Na literatura atual sobre as relações entre a alimentação, saúde e prevenção de doenças não transmissíveis destaca-se, precisamente, o interesse exponencial em examinar a alimentação humana numa perspectiva multidimensional, por oposição à visão direcionada para apenas um nutriente, alimento, ou mesmo, grupo de alimentos. 8 Neste contexto, o questionário de frequência alimentar (QFA) tem sido considerado uma referência nos estudos epidemiológicos ao que se refere a avaliação do consumo alimentar 9 e, especialmente, à ingestão nutricional, nomeadamente para avaliar a relação causal entre a alimentação e a ocorrência de desfechos clínicos como o aparecimento de doenças crônicas não transmissíveis. 10

O presente estudo teve como objetivo verificar se existem diferenças importantes em alguns aspectos nutricionais entre dois grupos de gestantes acompanhadas em serviços de atenção pré-natal, na região metropolitana e em uma pequena cidade do interior do Estado de Minas Gerais, Brasil.

## Métodos

O presente trabalho teve a participação de 240 gestantes, em idade entre 18 a 40 anos, em atendimento pré-natal no Ambulatório Jenny Faria, em Belo Horizonte (BH) e Posto de Saúde Padre Antônio Mendes, situado na cidade de Paula Cândido (PC), MG. As gestantes foram divididas em grupos, de acordo com cada trimestre, sendo 30 gestantes por trimestre na cidade do interior e 50 por trimestre na região metropolitana, totalizando 90 gestantes acompanhadas em Paula Cândido e 150 em Belo Horizonte. O quantitativo amostral foi baseado a partir da metodologia de diversos estudos publicados. O trabalho apresentou modelo do tipo transversal, havendo um contato com cada gestante.

Foi aplicado um questionário de frequência alimentar(QFA) contendo informações como idade, cor, escolaridade, estado civil, trabalho fora de casa e renda mensal. Além disto, foram aferidos o peso e estatura atual, assim como questionado o peso habitual anterior à gestação (peso pré-gestacional). Caso a gestante não se lembrasse, foi utilizado o primeiro peso no cartão da gestante, posteriormente foi calculado o IMC (índice de massa corpórea) pré-gestacional e atual.

Em relação à alimentação foram analisadas questões como: quantas refeições fazem ao dia, se tem costume de tomar café (com açúcar) toda hora, óleo utilizado nas refeições e consumo de refrigerante.

O QFA foi dividido em grupos alimentares e considerando os hábitos regionais. Utilizou-se como referência o questionário específico para gestantes, validado por Giacomello et al. 11 Não foi utilizado lista de alimentos, como proposto pelo autor. Já que este é um trabalho qualitativo e visa conhecer a alimentação habitual das gestantes de acordo com a pirâmide alimentar. O QFA foi dividido nas seguintes frequências de consumo: todos os dias, 5 a 6 dias por semana, 3 a 4 dias por semana, 1 a 2 dias por semana, 1 a 3 vezes por mês e raramente/nunca. Foram incluídas a frequência de quantas vezes almoça ou janta fora de casa; troca almoço e jantar por lanche; consome frutas; consome salada de vegetais crus; salada de vegetais cozidos (exceto batata, mandioca, inhame); consome carne; consome frituras; se utiliza gordura de porco nas refeições e qual frequência; consumo de leite; consumo de café; consumo de pão, biscoito ou bolo; produtos de padaria integral; arroz, macarrão; angu; *fastfood* ; alimentos ricos em açúcares e/ou gorduras; refrigerante e doces.

Os critérios de exclusão para o estudo foi a presença de diabetes ou outras doenças endócrinas, eclâmpsia ou pré-eclâmpsia, anemia ou outra deficiência nutricional previamente detectada em exame, além de vegetarianismo e intolerância a glúten e/ou lactose, pois poderia influenciar no hábito alimentar.

A ingestão alimentar habitual foi avaliada levando em consideração as porções recomendadas para cada grupo de alimentos na Pirâmide Alimentar Adaptada para gestantes 2 e foi utilizado o parâmetro recomendado pelo Instituto de Medicina 12 para ganho de peso em gestantes, de acordo com o trimestre.

Em relação à análise estatística, foi realizado uma análise descritiva para as variáveis quantitativas e qualitativas, com o uso de medidas de tendência central (usou-se o teste t para comparação de médias, uma vez que as suposições de normalidade foram garantidas).

Em um segundo momento, foi realizado uma comparação entre região metropolitana e interior, relacionando o ganho de peso com as variáveis de alimentação. A comparação foi feita de forma inferencial, utilizando o teste qui-quadrado de Independência para avaliar relação do ganho de peso com a alimentação, diferenciando para cada região. O nível de significância para todos os testes foi de 5%, e utilizado o programa SPSS versão 15.0.

## Resultados

**Fig. 1:**
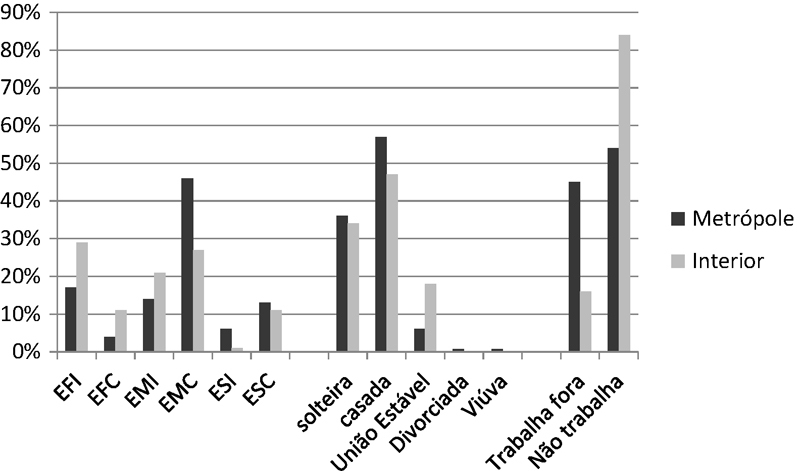
Características socioeconômicas das gestantes entrevistadas, nas regiões metropolitana e interior. Abreviações: EFI, ensino fundamental incompleto; EFC, ensino fundamental completo; EMI, ensino médio incompleto; EMC, ensino médio completo; ESI, ensino superior incompleto; ESC, ensino superior completo.

Em relação às variáveis socioeconômicas, em ambos os grupos, houve predomínio das gestantes que são casadas (BH = 56,6%; PC = 46,6%) e não trabalham fora de casa (BH = 54,6%; PC = 84,4%), a variável escolaridade apresentou maior porcentagem de ensino médio completo na região metropolitana (46%) e ensino fundamental incompleto na região interior (28,8%) ( Fig. 1 ).

A comparação das médias entre interior e região metropolitana foi significante no trimestre 2 para as variáveis idade ( *p* = 0,002), peso pré-gestacional ( *p* = 0,005), peso atual ( *p* = 0,0106) IMC pré-gestacional ( *p* = 0,02) e IMC atual ( *p* = 0,04). No 3° trimestre houve significância apenas para variável peso atual ( *p* = 0,03) ( Tabela 1 ).

**Tabela 1:** Comparação das médias, por região, das variáveis idade, peso pré gestacional, peso atual, altura, IMC pré gestacional e IMC atual

Trimestres	Variáveis	Região						Valor *p*
		Metropolitana			Interior			
		N	Média	Desvio padrão	N	Média	Desvio padrão	
Trimestre 1	Idade	50	29,0800	6,9776	30	27,1333	6,8619	0,2278
	PPG	50	66,7100	17,2069	30	65,8448	15,2434	0,8210
	PA	50	69,6410	19,2132	30	68,0500	16,5144	0,7069
	Altura	50	1,6180	0,0777	30	1,6097	0,0544	0,5755
	IMCPG	50	27,6212	18,2511	30	25,7757	5,5977	0,5923
	IMCA	50	28,6178	17,6039	30	26,2210	5,8661	0,4734
Trimestre 2	Idade	50	28,0800	5,7812	30	24,2000	4,2051	0,0020
	PPG	50	65,8160	18,6135	30	56,9700	8,5317	0,0050*
	PA	50	70,7380	19,2241	30	62,2983	9,4756	0,0106*
	Altura	50	1,6120	0,0745	30	1,5830	0,0684	0,0862
	IMCPG	50	25,2588	6,7911	30	22,7197	2,9428	0,0239*
	IMCA	50	27,1560	6,9664	30	24,8427	3,2956	0,0487*
Trimestre 3	Idade	50	27,7600	6,9063	30	26,2069	7,1733	0,3401
	PPG	50	67,0320	14,1164	30	61,7733	13,5451	0,1056
	PA	50	77,7320	13,0585	30	71,2667	13,5195	0,0376*
	Altura	50	1,6184	0,0604	30	1,5953	0,0604	0,1023
	IMCPG	50	25,6312	5,4437	30	24,4700	6,2182	0,3840
	IMCA	50	29,7092	4,9372	30	28,1977	6,3400	0,2377

Abreviações: IMCPG, índice de massa corporal pré-gestacional; IMCA, índice de massa corporal atual; PA, peso atual; PPG, peso pré-gestacional.

Teste x ^2^ : associação significativa para*p < 0,05.

A frequência das refeições diárias obtiveram predomínio de 3 a 4 refeições por dia no 1° e 2° trimestres (BH = 54,0 e 46%, respectivamente; PC = 66,7 e 63,3%, respectivamente), exceto entre as gestantes do 3° trimestre de BH, que consumiam 5 a 6 refeições ao dia (44%; *n* = 22). O ganho de peso foi dividido por trimestre e calculado o ganho ponderal significativo ou não, baseado nas recomendações propostas pelo IOM. 12 Portanto, foram classificadas como “não houve ganho de peso” as gestantes que estavam dentro da faixa aceitável a partir do seu estado nutricional pré-gestacional. Sendo assim, de acordo com os resultados, houve ganho de peso somente no primeiro trimestre, nas regiões metropolitana e interior (58,0 e 53,3%, respectivamente) ( Tabela 2 ).

**Tabela 2:** Número de refeições realizadas por dia e ganho de peso * acima do recomendado, por trimestre gestacional e região

Trimestre Gestacional			Região Metropolitana		Interior		Total
			n	Fr (%)	n	Fr (%)	
Trimestre 1	Faixa do n° de refeições	1 a 2 refeições	2	4	3	10	5
		3 a 4 refeições	27	54	20	66,7	47
		5 a 6 refeições	14	28	7	23,3	21
		> 6 refeições	7	14	0	0	7
	Total		50	100	30	100	80
	Ganho de peso	Não houve ganho	21	42	14	46,7	35
		Houve ganho	29	58	16	53,3	45
	Total		50	100	30	100	80
Trimestre 2	Faixa do n° de refeições	1 a 2 refeições	1	2	4	13,33	5
		3 a 4 refeições	23	46	19	63,33	42
		5 a 6 refeições	20	40	7	23,33	27
		> 6 refeições	6	12	0	0	6
	Total		50	100	30	100	80
	Ganho de peso	Não houve ganho	38	76	24	80	62
		Houve ganho	12	24	6	20	18
	Total		50	100	30	100	80
Trimestre 3	Faixa do n° de refeições	1 a 2 refeições	2	4	5	16,7	7
		3 a 4 refeições	17	34	16	53,3	33
		5 a 6 refeições	22	44	8	26,7	30
		> 6 refeições	9	18	1	3,3	10
	Total		50	100	30	100	80
	Ganho de peso	Não houve ganho	35	70	24	80	59
		Houve ganho	15	30	6	20	21
	Total		50	100	30	100	80

* O cálculo do ganho de peso foi realizado a partir das recomendações do Instituto de Medicina (1990), de acordo com o peso pré gestacional. IMC pré gestacional (<19,8): baixo peso. Ganho de peso de 2,3kg no 1° trimestre e ganho total entre 12,5–18kg; IMC pré gestacional (19,8–26,0): adequado. Ganho de peso de 1,6kg no 1° trimestre e ganho total entre 11,5–16kg; IMC pré gestacional (26,0–29,0): sobrepeso. Ganho de peso de 0,9kg no 1° trimestre e ganho total entre 7–11,5kg; IMC pré gestacional (>29,0): obesidade. Manter o peso pré gestacional 1° trimestre e ganho total de 7kg.

Posteriormente, comparou se o ganho de peso é influenciável ou não por diversos hábitos alimentares. Houve diferença para as variáveis “almoça ou janta fora de casa,” no 1° trimestre para as gestantes de BH ( *p* = 0,006); “quantas vezes consome leite,” no 1° trimestre do grupo do interior ( *p* = 0,03); “quantas vezes consome *fastfood* ,” no 3° trimestre das gestantes de BH ( *p* = 0,009) ( Tabela 3 ). *

**Tabela 3:** Hábitos alimentares e sua relação com o ganho de peso dentro do recomendado (valores significativos)**

Trimestre Gestacional		Região							
		Metropolitana				Interior			
		Ganho de peso		Total	Valor *p*	Ganho de peso		Total	Valor *p*
	Quantas vezes almoça ou janta fora	Não houve ganho	Houve ganho			Não houve ganho	Houve ganho		
Trimestre 1	Raramente/nunca	9	15	24	0,006*	5	10	15	0,099
	1–3 vezes/mês	0	7	7		2	4	6	
	1–2 dias	7	4	11		6	1	7	
	3–4 dias	0	2	2		1	0	1	
	5–6 dias	0	1	1		0	1	1	
	Todos os dias	5	0	5		0	0	0	
	Total	21	29	50		14	16	30	
Trimestre 1	Quantas vezes consome leite	Ganho de peso		Total	Valor p	Ganho de peso		Total	Valor *p*
		Não houve ganho	Houve ganho			Não houve ganho	Houve ganho		
	Raramente/Nunca	5	2	7	0,183	1	1	2	0,030*
	1–3 vezes/mês	2	1	3		0	1	1	
	1–2 dias	4	3	7		5	1	6	
	3–4 dias	2	1	3		4	0	4	
	5–6 dias	1	3	4		1	3	4	
	Todos os dias	7	19	26		3	10	13	
	Total	21	29	50		14	16	30	
Trimestre 3	Quantas vezes consome *fastfood*	Ganho de peso		Total	Valor p	Ganho de peso		Total	Valor *p*
		Não houve ganho	Houve ganho			Não houve ganho	Houve ganho		
	Raramente/nunca	19	15	34	0,009*	15	4	19	0,682
	1–3 vezes/mês	8	0	8		5	0	5	
	1–2 dias	0	3	3		4	1	5	
	3–4 dias	4	0	4		0	0	0	
	5–6 dias	1	0	1		1	0	1	
	Todos os dias	0	0	0		0	0	0	
	Total	32	18	50		25	5	30	

Teste x ^2^ *p < 0,05 hábito alimentar *versus* ganho de peso.

**Tabela completa, contendo os valores não significativos em anexo.

## Discussão

Um resultado importante encontrado no estudo foi a prevalência de sobrepeso no período pré-gestacional na região metropolitana, já que todas as médias de IMC se enquadraram na faixa de sobrepeso. Além disto, foi observado ganho de peso significativo somente no primeiro trimestre em ambas as regiões, no segundo e terceiro trimestres a maioria das gestantes obtiveram ganho de peso dentro do recomendado.

A gestação pode atuar como desencadeante da obesidade ou como agravante, quando esta for preexistente. Na avaliação do estado nutricional durante período da gestação, houve um aumento no sobrepeso quando comparados com o período pré-gestacional, semelhantes a outros estudos. 13 Em relação ao período gestacional, o maior risco para complicações relacionadas ao ganho de peso inadequado são para as gestantes obesas, que estão suscetíveis a diabetes, hipertensão, parto cirúrgico, enquanto seus filhos são mais propensos a apresentarem microssomia, riscos de malformação fetal e maior mortalidade perinatal. 14

O estado nutricional pré-gestacional é um dos principais fatores associados ao ganho de peso durante a gravidez. 15 A chance elevada de ganho excessivo de peso em gestantes com sobrepeso e obesidade pré-gestacional confirma os achados de duas coortes, uma com 141 gestantes saudáveis de um serviço público do município de São Paulo 16 e outra com 667 gestantes acompanhadas até o parto na rede básica de saúde do Estado do Rio Grande do Sul. 17

Em relação às variáveis socioeconômicas, notamos que o nível de escolaridade é menor no interior e, além disto, menos gestantes se encontram inseridas no mercado de trabalho. A literatura refere que condições socioeconômicas desfavoráveis produzem resultados insatisfatórios na saúde da população em geral, e quanto maior a renda, maior o poder de compra e acesso a alimentação variada, 18 o que pode influenciar na saúde materna e fetal. A baixa escolaridade pode ser vista como agravante na saúde das mulheres, pois é considerado pelo Ministério da Saúde como fator de risco obstétrico. 19 Apesar da maioria das gestantes entrevistadas não ter alta escolaridade no interior, houve busca pelo serviço de saúde para do pré-natal, o que pode revelar uma tendência ao interesse pela sua saúde e de seus filhos.

Como dito anteriormente, foi observado nas gestantes de BH uma tendência a sobrepeso e maior nível de escolaridade, tal como em estudo realizado em São Paulo, em que gestantes com quatro anos ou mais de estudo apresentaram ganho de quase dois quilos a mais quando comparadas às demais, sendo a escolaridade considerada um marcador de acesso aos alimentos. 6 Por outro lado, em Recife, a baixa escolaridade contribuiu para o ganho de peso excessivo, o nível de escolaridade reflete a situação socioeconômica, sendo assim, as gestantes com menor poder aquisitivo priorizam o consumo de alimentos mais calóricos (ricos em gorduras, particularmente de origem animal, açúcar e alimentos refinados, em detrimento aos carboidratos complexos e fibras) por terem menor custo. 20

O número de refeições diárias é menor que o preconizado por Accioly et al, 21 que recomenda 5 a 6 refeições por dia. A dieta deve ser fracionada com menores porções (café da manhã, lanche - opcional, almoço, merenda, jantar, ceia – opcional com intervalo de 3/3h) para que ao longo do dia, haja aporte de nutrientes e energia necessários. No entanto, o estudo demonstrou predominância de 3 a 4 refeições por dia, estando adequado somente na região metropolitana no terceiro trimestre. A análise do consumo alimentar não demonstrou grande discrepância com o recomendado pela pirâmide alimentar. Houve prevalência no consumo diário de frutas, verduras e vegetais, leguminosas, cereais, leite e carnes e baixo consumo de *fastfood* , alimentos ricos em açúcares e/ou gorduras e refrigerante. Isto pode ser consequência do fato de uma nutricionista aplicar os questionários, levando as gestantes a informar uma alimentação saudável, já que as mesmas possuem informações sobre alimentação saudável, porém, nem sempre as colocam em prática. 22

Uma limitação do estudo foi a utilização do QFA como método de avaliação do consumo alimentar, uma vez que ele contém grupos de alimentos, limitando uma comparação com história dietética. 23

Estudos recentes demonstram que alimentos processados e industrializados tem sido identificado entre as gestantes em diversas regiões do mundo e reflete as mudanças ocorridas no mundo moderno, como a busca por maior comodidade, praticidade e rapidez, que afetam diretamente a alimentação da população. 24 25 26 27 No Brasil, similarmente às demais regiões do mundo, essas modificações estão relacionadas com alterações no estilo de vida, que se expressam pela redução do gasto calórico diário e na adoção de hábitos alimentares caracterizados por elevado consumo de gorduras saturadas, de açúcares simples, produtos industrializados e redução no consumo de frutas, verduras e legumes. Este padrão de consumo alimentar comprova a tendência de modificação dos hábitos alimentares registrados por pesquisas ao longo do tempo. 28

Nota-se prevalência de consumo de frutas, leite e salada de vegetais crus ou cozidos (folhas, leguminosas, raízes, etc). Estes hábitos contribuem com a adequação de proteínas e carboidratos simples e complexos e dos micronutrientes (vitaminas e minerais). Assim, podem estar associados a melhores condições de saúde da gestante e ao crescimento e desenvolvimento fetal. Deve-se salientar que esses padrões apresentam em suas composições nutrientes essenciais para a saúde humana e são considerados os mais próximos das recomendações dietéticas emanadas atualmente para gestação e como tal, pode ser o mais intuitivamente associado às melhores condições de saúde na gestação, ao crescimento e desenvolvimento adequado do feto. 29 Em estudo realizado por Belarmino et al 30 foi investigado o consumo alimentar de acordo com os grupos de alimentos que compõem a pirâmide alimentar, sendo categorizados nos seis grupos de alimentos: grupo A (pão, arroz, cereais e massas); grupo B (vegetais e frutas); grupo C (carnes, aves, peixes e ovos); grupo D (feijão e nozes); grupo E (leite, iogurte e queijo); e grupo F (gorduras, óleos e doces). No trabalho supracitado, verificou-se o baixo consumo de alimentos do grupo B, diferente do que foi encontrado no presente estudo.

Muito ainda há que ser feito para que mais gestantes atentem para educação nutricional satisfatória. Só assim, elas poderão compreender melhor o período e as mudanças que estão vivenciando e poderão praticar hábitos alimentares saudáveis.

O presente estudo permitiu concluir que as gestantes da região metropolitana e interior, de modo geral, se alimentam de maneira adequada, com hábitos alimentares diversificados e favoráveis à manutenção da saúde, rico em frutas e verduras. Porém, também se verificou um perfil sociodemográfico pouco favorável ao período gestacional, com baixa escolaridade e renda das entrevistadas, principalmente no interior. Sugere-se que estudos mais detalhados sejam realizados, verificando a associação entre o perfil alimentar da gestante e a saúde do recém-nascido, para que seja possível identificar os fatores de risco gestacionais para a saúde da mãe e do concepto.

## Footnotes

* A tabela completa, com todas as informações e valores de significância podem ser vistas em material suplementar (rbgo.com.br).
